# Validating self-report of diabetes use by participants in the 45 and up study: a record linkage study

**DOI:** 10.1186/1472-6963-13-481

**Published:** 2013-11-19

**Authors:** Elizabeth Jean Comino, Duong Thuy Tran, Marion Haas, Jeff Flack, Bin Jalaludin, Louisa Jorm, Mark Fort Harris

**Affiliations:** 1 Centre for Primary Health Care and Equity, University of New South Wales, Sydney, NSW 2052, Australia; 2 Centre for Health Research, School of Medicine, University of Western Sydney, Locked Bag 1797, Penrith, NSW 2751, Australia; 3 Centre for Health Economics Research and Evaluation, Faculty of Business, University of Technology, Sydney, PO Box 123, Broadway, NSW 2007, Level 4, 645 Harris Street, Ultimo, NSW 2007, Australia; 4 Diabetes Centre, Bankstown-Lidcombe Hospital, Eldridge Road, Bankstown, NSW 2200, Australia; 5 Centre for Research, Evidence Management and Surveillance, Sydney and South Western Sydney Local Health Districts, Locked Bag 7017, Liverpool, NSW 1871, Australia; 6 School of Public Health and Community Medicine, University of New South Wales, Sydney 2052, Australia

**Keywords:** Primary health care, Cohort studies, Diabetes mellitus, Record linkage, Health service data, Quality of health care, Validation study, Sensitivity and specificity, Older age, English language

## Abstract

**Background:**

Prevalence studies usually depend on self-report of disease status in survey data or administrative data collections and may over- or under-estimate disease prevalence. The establishment of a linked data collection provided an opportunity to explore the accuracy and completeness of capture of information about diabetes in survey and administrative data collections.

**Methods:**

Baseline questionnaire data at recruitment to the 45 and Up Study was obtained for 266,848 adults aged 45 years and over sampled from New South Wales, Australia in 2006–2009, and linked to administrative data about hospitalisation from the Admitted Patient Data Collection (APDC) for 2000–2009, claims for medical services (MBS) and pharmaceuticals (PBS) from Medicare Australia data for 2004–2009. Diabetes status was determined from response to a question ‘*Has a doctor EVER told you that you have diabetes*’ (n = 23,981) and augmented by examination of free text fields about diagnosis (n = 119) or use of insulin (n = 58). These data were used to identify the sub-group with type 1 diabetes. We explored the agreement between self-report of diabetes, identification of diabetes diagnostic codes in APDC data, claims for glycosylated haemoglobin (HbA1c) in MBS data, and claims for dispensed medication (oral hyperglycaemic agents and insulin) in PBS data.

**Results:**

Most participants with diabetes were identified in APDC data if admitted to hospital (79.3%), in MBS data with at least one claim for HbA1c testing (84.7%; 73.4% if 2 tests claimed) or in PBS data through claim for diabetes medication (71.4%). Using these alternate data collections as an imperfect ‘gold standard’ we calculated sensitivities of 83.7% for APDC, 63.9% (80.5% for two tests) for MBS, and 96.6% for PBS data and specificities of 97.7%, 98.4% and 97.1% respectively. The lower sensitivity for HbA1c may reflect the use of this test to screen for diabetes suggesting that it is less useful in identifying people with diabetes without additional information. Kappa values were 0.80, 0.70 and 0.80 for APDC, MBS and PBS respectively reflecting the large population sample under consideration. Compared to APDC, there was poor agreement about identifying type 1 diabetes status.

**Conclusions:**

Self-report of diagnosis augmented with free text data indicating diabetes as a chronic condition and/or use of insulin among medications used was able to identify participants with diabetes with high sensitivity and specificity compared to available administrative data collections.

## Background

Surveys and questionnaires are frequently used in health research to collect information about socio-demographic characteristics, functional health status, access to health care, and presence of lifestyle and other risk factors [1-5]. The reliability, validity and consistency of self-report of health status and receipt of health care have been issues of concern for health researchers. Under-reporting of conditions may occur because people have not been formally diagnosed, or chose not to reveal this information, or do not relate the condition to the particular situation or timeframe in which the information was sought. Some studies have found that the reliability of reporting is better for conditions where there are clear diagnostic criteria such as diabetes than for conditions where the diagnostic criteria are less clear such as chronic obstructive pulmonary disease [6]. Reliability of reporting can also be influenced by personal characteristics such as age and gender [6]. Prevalence estimates based on self-report under-enumerate actual population rates [2,4]. This was highlighted by the Australian Diabetes, Obesity and Lifestyle Study (AusDiab) study which reported that only approximately half of adults who participated in clinical testing for diabetes and subsequently were diagnosed with diabetes actually reported a prior diagnosis [2].

Administrative data collections represent alternative sources of information on health status and access to health care [6]. Although these exist primarily for the purposes of managing and operating health services, they are increasingly used in health services research. In Australia, there are four main sources of administrative data for diabetes: the National Diabetes Services Scheme (NDSS), Medicare Australia data and hospital morbidity data collections. The NDSS is a voluntary support services for Australians with diabetes and provides access to subsidised diabetes management products and services. Registration is through general practice and coverage of up to 80-90% of Australians with diabetes is reported. Medicare Australia is the country’s universal health insurance scheme and administers claims for subsidised medical care under the Medical Benefits Schedule (MBS) schedule and for pharmaceutical products under the Pharmaceutical Benefits Schedule (PBS). These claim data may under- or over-estimate diabetes care provision and diabetes prevalence due to subsidy rules. For example, claims for pathology testing recorded in MBS data are limited to a maximum of four tests in a patient episode for a set of pathology services, ordered by a general practitioner for a non-hospitalised patient [7]. Among a battery of tests, this ‘coning’ will limit claims to the four most expensive tests and may under-capture claims for HbA1c tests. Over-estimation based on HbA1c testing may also occur if the test is being used for other purposes such as screening. Dispensed medications with prices lower than the general patient co-payment ($34.20 as at 2011) or private scripts are not captured in PBS data. In New South Wales (NSW) inpatient data are recorded in the Admitted Patient Data Collection (APDC), and these data include details about episodes of care for people who are admitted. However, according to Australian Coding Standard for Additional Diagnoses (ACS0002) the recording of diabetes as an additional diagnosis is only required if diabetes affects patient management during hospital admission [8]. Population data that includes diagnostic testing such as AusDiab data rarely exists [9].

Thus variable estimates of prevalence occur due to uneven population coverage, the age group reported, currency of the data source, and frequency of updates to the data source. The prevalence of diabetes for all ages based on self-reported data from Australia’s National Health Survey was 3.3% in 2004-5 [10] and from the South Australian Omnibus Survey in 2003 among people aged 15 years or older was 6.7% [5]. Prevalence rates based on administrative data collected during 2004–5 including NDSS (3.6%), MBS (3.0%) and PBS (3.0%) were similar [10]. Although somewhat old now (1999–2000) the Australian Diabetes and Lifestyle Study baseline data reported a self-reported prevalence of 3.7% and further undiagnosed (following testing) prevalence of 4.2% in adults aged 25 years or more [2]. Baseline data from the 45 and Up Study of NSW residents aged 45 years or older found a prevalence of 8.8% [11].

Record linkage provides an opportunity to explore the accuracy and completeness of capture of information about diabetes in survey and administrative data collections. We were unable to identify other studies comparing self-reported diabetes status with administrative data collections. Studies that validate quality of care measures suggest that patients overestimate testing procedures compared to medical records [12,13]. This paper reports a record linkage study using self-reported data from a baseline questionnaire completed at recruitment to the 45 and Up Study linked to record extracts from administrative data collections including MBS, PBS and APDC. The baseline data provided complete enumeration of the cohort whereas not every cohort participant was identified in each of the administrative data collections. The aim of this study is to compare and contrast indicators of prevalent diabetes among the linked data. A secondary aim was to test an algorithm to differentiate type 1 and type 2 diabetes. Specifically we wanted to demonstrate the value of self-report of diabetes status in establishing a diabetes cohort using record linkage. This is a part of a larger study which is investigating the relationship between primary health care and health outcomes in people with diabetes.

## Methods

### Study population

The 45 and Up Study is a population-based cohort study of NSW residents aged 45 years and older. It is described in detail elsewhere [11]. Briefly, recruitment was undertaken between 2006 and 2009. Potential participants were randomly selected from the Medicare Australia database (Australia’s universal public health insurance system). Participants joined the Study by completing a mailed self-administered questionnaire and providing consent for long term follow-up, including linkage to personal health records. The response rate was 18% [11]. This current study used baseline questionnaire data from 266,848 participants.

### Detection of diabetes status and diabetes medication from baseline questionnaire

The questionnaire completed at recruitment is available at http://www.saxinstitute.org.au/ourwork/45-up-study/questionnaires. The participants could indicate their diabetes status through their responses to three questionnaire items. Most participants with diabetes were identified from tick box responses to Question 24: ‘*Has a doctor EVER told you that you have diabetes?*’. Participants who responded affirmatively to this question were asked to indicate ‘*Age when condition was first found’*. Participants could also indicate a diagnosis of diabetes in a free-text box response to Questions 26: ‘*Are you NOW suffering from any other important illness*?’ The SAS Perl regular expression function [14] which matches and allocates text patterns in string variables was used to search for mention of diabetes among data from participants who did not report a diagnosis of diabetes using the tick box in Question 24. Free-text box responses containing ‘diabetes-like’ keywords were manually examined to identify additional participants with diabetes. These participants were categorised as having diabetes if they wrote diabetes-specific text such as diabetes, sugar diabetes, diabetes type 1, diabetes type 2, grade 2 diabetes, self-management or controlled diabetes. Participants writing text such as pre-diabetes, borderline diabetes, or diabetes insipidus were categorised as ‘diabetes uncertain’.

The self-reported use of diabetes medications was also identified from the baseline questionnaire. Question 23 asked ‘*Have you taken any medications, vitamins or supplements for most of the last 4 weeks, including HRT and the pill*?’ Medications for diabetes could be identified from the tick box for Diabex, Diaformin, or Metformin, and a text box for other diabetes related medications. A list of the different types and brand names of insulin products and oral hypoglycaemic agents (OHA) available in Australia was accessed [15] (Table 1) and searched, using the SAS Perl function [14] (false negative rate of 1.7% for insulin and OHAs, false positive rates for insulin and OHAs ranged between 0% and 1.06%). Participants who reported use of insulin but did not indicate a diagnosis of diabetes in other fields (n = 58) were added to the diabetes group while those who reported only OHAs were included in the ‘diabetes uncertain’ group. We did not attempt to identify incident cases of diabetes.

**Table 1:** Insulin products and oral hypoglycaemic agents (OHA) available in Australia and self-reported use by 45 and Up Study participants at recruitment

**Medications self-reported**	**Number**
Insulin	2,379
Any oral hypoglycaemic agents (OHAs)	14,124
Biguanides	12,560
Sulphonylureas	3,766
Thiazolidinediones	855
Alpha glucosidase inhibitor	72
Inhibitor DipeptidylPeptisase 4 inhibitor	50
Pre-mixed tablets	196
Exenatide	17
Meglitinides	6

Finally, we attempted to classify diabetes as type 1, type 2, or other based on additional information including age at diagnosis, medication use, and age at birth of their last child (for females). Participants who either reported type 1 diabetes in free-text fields, were diagnosed before age of 31 years [16] and were using insulin, or did not give age of diagnosis but were using insulin were classified as having type 1 diabetes. Women who were diagnosed before the date of last delivery and did not report current medication use were classified as having (had) gestational diabetes. The remainder were classified as having type 2 diabetes.

### Validation of self-report diabetes status

Diabetes status identified from the 45 and Up questionnaire was compared with diabetes status reported in the APDC, and MBS and PBS claims using record linkage. The **APDC** is a mandated record of all patient admissions to NSW hospitals. Data between 1 July 2000 and 31 December 2009 were extracted. Patient diagnoses are coded as principal diagnosis and additional (>10) diagnoses using ICD10-AM codes [8]. Diabetes diagnoses in the APDC were identified from all diagnostic fields using ICD10-AM codes (E10, E11, E13, E14, and O24.0-O24.3) and were classified as Type 1, Type 2, and other type of diabetes. The 45 and Up Study data and APDC were linked using ‘best practice protocols’ for preserving individual privacy by the Centre for Health Record Linkage (CHeReL), an independent data linkage facility [17]. Personal information such as name, date of birth, sex and address was used for linkage.

The **MBS data** records all claims for medical and diagnostic services provided through Medicare. Claims for glycosylated haemoglobin (HbA1c) testing (item number 66551) between 2004 and 2009 were extracted. HbA1c testing is used as a measure of glucose control for people with diabetes and should be performed at least annually for all people with diabetes [15]. Medicare data were linked by Department of Human Services using deterministic techniques based on a unique identifying number and other personal identifying information.

### Validation of self-report diabetes medications

Self-reported medication use was compared with available claims for dispensing of insulin and oral hypoglycaemic agents (OHA) products recorded in the PBS data for 12 months prior to recruitment. Medications provided to war veterans are not included in the PBS, so participants who reported possessing a Department of Veteran Affairs (DVA) card were excluded from this analysis (n = 6,303). (OHA use was tested in concession card holders only).

### Analysis

As described above, participants were categorised into one of three diagnostic groups: diabetes, non-diabetes, and diabetes uncertain. Data from participants in the ‘uncertain’ group were analysed separately.

The diabetes and non-diabetes groups were compared to the criterion standard of each linked data collection. The criterion standards used were: confirmed having diagnosis of diabetes in diabetes ICD-10-AM codes recorded in APDC data, a claim for (1 or 2) HbA1c tests in MBS data and a claim for a dispensed prescription for insulin or OHA in the PBS data. We constructed two by two tables and calculated sensitivity, specificity, positive predictive value (PPV), negative predictive value (NPV), and Kappa agreement. Using administrative data as the imperfect ‘gold’ standard, sensitivity refers to the proportion of participants with diabetes according to criterion standard of each linked administrative data collection who were assigned to the diabetes group, whereas specificity refers to the proportion of participants without diabetes according to criterion standard of each linked administrative data collection who were not assigned to the diabetes group [18]. The PPV refers to the proportion of participants in the 45 and Up Study assigned to the diabetes group who were also found to have diabetes according to the relevant criterion standard. The NPV is the proportion of participants who were assigned to the non-diabetes group without diabetes according to the criterion standard. The Kappa statistic provides an error-corrected measure of agreement between two measures of a categorical variable without assuming either source as the gold standard. It was calculated using Fleiss’ formula [19].

### Ethics

The 45 and Up Study was approved by the University of New South Wales Human Research Ethics Committee. The specific study reported here was approved by the NSW Population and Health Services Ethics Committee, Cancer Institute NSW (reference 2010/05/228 and 2010/05/229).

## Results

### Report of diabetes status from baseline 45 and Up questionnaire

**Figure 1:**
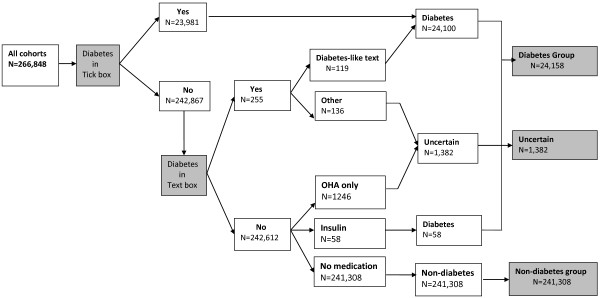
Algorithm for identifying diabetes status among participants at recruitment using information reported during completion of a baseline questionnaire.

Data provided by 266,848 NSW residents at recruitment to the 45 and Up Study were extracted. The distribution of diabetes is summarised in Figure 1. A prior diagnosis of diabetes was reported by 23,981 (9.0%) participants. On examination of the ‘free text’ field, a further 255 participants wrote diabetes related words, 119 of whom were considered to have current diabetes and 136 whose diabetes status was uncertain. Examination of the medication use fields for the remaining 242,612 participants identified 1,246 participants taking OHAs and 58 using insulin. Participants who were using insulin were included in the diabetes group, while those in the OHA group who did not tick the diabetes diagnosis box were assigned to diabetes uncertain group. In total 24,158 individuals were assigned to the diabetes group, giving a revised reported prevalence of diabetes of 9.05%.

### Validation of self-reported diabetes status from the 45 and Up Study

#### Validation against APDC diagnostic codes

There were 193,126 (19,086 in the diabetes group, and 153 uncertain) participants in the 45 and Up Study with an identified hospital admission between 2000 and 2009. 45 and Up Study participants could not be linked to data held by the CHeReL (n = 77), were not admitted to hospital during study period (n = 72,953), or were diagnosed with diabetes since their last hospital admission (n = 692) were excluded from this analysis. Participants with a diagnosis of gestational diabetes and not other forms of diabetes in the APDC data were excluded from further analysis.

Among 45 and Up Study participants assigned to the diabetes group (n = 19,086), 79.3% (n = 15,143) had a diagnosis of diabetes recorded in APDC (PPV); among participants assigned to the non-diabetes group, 98.3% (n = 169,983) did not have diabetes recorded in the APDC (NPV, Table 2). Using APDC as the standard the sensitivity was 83.9%, specificity 97.7%, and Kappa for agreement was 0.8. About half of those assigned to the diabetes uncertain group at baseline (n = 557; 48.3%) had diabetes recorded in the APDC.

**Table 2:** Agreement between self-report of diabetes status by 45 and Up Study participants at recruitment and at least one diagnostic code for diabetes reported in APDC data 2000–9

**Self-report of diabetes**	**APDC diabetes status**		**Total**
	**Diabetes**	**Non-diabetes**	
Diabetes	15,143	3,943	19,086
Non-diabetes	2,904	169,983	172,887
Total	18,047	173,926	191,973^#^
Uncertain*	557	596	1153

* Not included in the agreement analyses.

^#^ Data limited to participants with record of hospital admission during 200–9.

#### Validation of diabetes against claims for HbA1c testing in MBS data

Of the 266,848 45 and Up Study participants, 265,052 successfully linked to MBS data (including 24,004 in the diabetes group and 1,377 in the diabetes uncertain group). Participants whose data did not link to MBS (n = 1,783) or whose year of diagnosis with diabetes was later than 2009 (n = 13) were excluded from this analysis. During the five years 2005–09, 32,641 participants (12.3%) had at least one MBS claim for an HbA1c test; 22,467 (8.5%) had two claims; 17,663 (6.7%) had three claims; and 14,182 (5.4%) had at least four claims for an HbA1c test.

Table 3 presents the cross-tabulations between self-reported diabetes status from the 45 and Up Study and at least one claim for an HbA1c test. Among 45 and Up Study participants assigned to the diabetes group (n = 24,004), 84.7% (n = 20,340) had a claim for a HbA1c test recorded (PPV); among participants assigned to non-diabetes group, 95.2% (n = 228,157) did not have a claim for a HbA1c test recorded (NPV, Table 2) (73.4% and 98.2% respectively if two claims for HbA1c testing were considered). Using claims for HbA1c tests as the standard, the sensitivity was 63.9%, specificity was 98.4%, and Kappa was 0.7 (80.4%, 97.4%, and 0.75 respectively if two claims for HbA1c testing were considered). About half (n = 787; 57.2%) of those of uncertain diabetes status at recruitment had at least one record of an HbA1c test, suggesting presence of diabetes.

**Table 3:** Agreement between self-report of diabetes status by 45 and Up Study participants at recruitment and claims for at least 1 HbA1c test during 2005 - 2009

**Self-report of diabetes**	**HbA1c testing status**		**Total #**
	**yes**	**no**	
Diabetes	20,340	3,664	24,004
Non-diabetes	11,514	228,157	239,671
Total	31,854	231,821	263,675
Uncertain*	787	590	1,377

* Not included in the agreement analyses.

^#^ Data limited to participant data that linked successfully to MBS data.

#### Validation of self-reported medication use against claims for diabetes medication recorded in PBS data

Of the 266,848 participants in the 45 and Up Study, 213,287 participants linked to PBS data, did not report DVA card at baseline, and had at least one claim for medication during the 12 months prior to recruitment.

At baseline of 24,151 participants in the 45 and Up Study assigned to the diabetes group, 10% (n = 2,379) self-reported using insulin and 58% (n = 13,962) self-reported OHAs. In the linked data, 71.4% (n = 14,465) of participants with diabetes had a record of at least one PBS claim for diabetes-related medication during the 12 month prior to enrolment and 99.7% (191,068) of those assigned to the non-diabetes group did not have a claim recorded for diabetes-related medication.

Table 4 presents the cross-tabulations between self-reported diabetes status from the 45 and Up Study and at least one claim for insulin or OHA from the PBS. Using the PBS data as the standard the sensitivity was 95.6%, the specificity was 97.1% and the Kappa was 0.8. About two thirds (n = 770; 66.2%) of those whose diabetes status at baseline was uncertain has a claim for diabetes-related medication.

**Table 4:** Agreement between self-report of diabetes status by 45 and Up Study participants at recruitment and claims for diabetes related medication (Insulin and/or OHAs) in the 12 months prior to recruitment

**Self-report of diabetes**	**Diabetes related medication**		**Total #**
	**yes**	**no**	
Diabetes	14,465	5,780	20,245
Non-diabetes	663	191,068	191,731
Total	15,128	196,848	211,976
Uncertain*	770	394	1,164

* Not included in the agreement analyses.

^#^ Data limited to participants that linked successfully to PBS data and met inclusion criteria.

#### Determination of type of diabetes

**Figure 2:**
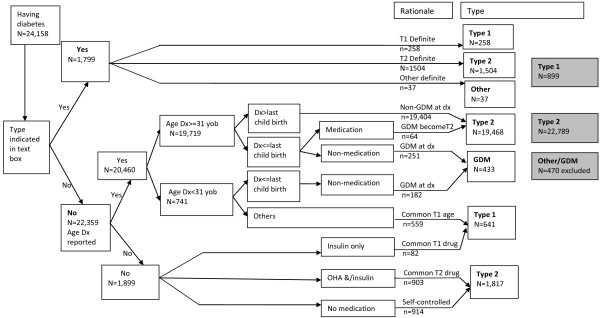
**Algorithm for classifying type of diabetes (Type1, Type 2, Other) for participants who reported diabetes at recruitment and using information reported during completion of a baseline questionnaire.** Footnote: data limited to 24,158 participants categorised to the diabetes group.

Our algorithm for determining the type of diabetes among the 24,158 participants assigned to the diabetes group is summarised in Figure 2. Using the free text field, 258 participants indicated that they had type 1 diabetes and 1,504 indicated that they had type 2 diabetes. Participants who reported using insulin products AND who reported diagnosis before age 31 (n = 559) or did not provide an age of diagnosis (n = 62) were categorised as type 1 diabetes. Women who gave their age of diagnosis before the birth of their last child and were not currently using diabetes medication were categorised as gestational diabetes. This process resulted in 899 participants categorised as type 1, 22,789 as type 2 and 470 as ‘other’.

Table 5 illustrates the agreement between diabetes type identified using our algorithm and diabetes type as indicated in the APDC data for participants with diabetes with a record of hospital admission. Among the 18,000 participants in the 45 and Up Study baseline data who were assigned to Type 2 diabetes using the algorithm, 76.8% had a diagnosis of type 2 diabetes recorded in the APDC data. However, among the 756 identified assigned to Type 1 diabetes using our algorithm, only 41.5% had a diagnosis of Type 1 diabetes recorded in the APDC data.

**Table 5:** Agreement between algorithm for assigning diabetes type based on baseline questionnaire data reported by 45 and Up Study participants and diabetes type recorded in APDC data 2000-9

**Diabetes type from baseline**	**Diabetes type – APDC**					**Total**
	**Type 1**	**Type 2**	**Gestational/other**	**Unspecified**	**Diabetes not recorded**	
Type 1	314	383	2	1	56	756
Type 2	451	13822	18	45	3664	18,000
Gestational/other	26	61	20	0	223	330
Total	791	14,266	40	46	3,943	19,086^#^

^#^ Data limited to 45 and Up participants with diabetes with a record of hospital admission during 200–9.

## Discussion

This study explores the criterion validity of identifying participants with diabetes from information they provided at recruitment to the 45 and Up Study. Most participants (99%) with diabetes self-reported their diabetes status through a tick box response; the remainder wrote diabetes in free text field (n = 119) or reported insulin use (n = 58). The final prevalence of diabetes was 9.05% (n = 24,158). Compared to the other data collections, more than 80% of those who self-reported diabetes were found to have a record of a diagnosis of diabetes or diabetes-related treatment in linked data collections. There were 1,382 participants (0.5%) whose diabetes status was uncertain based on self-reported information about their diabetes status. Although about half of this ‘diabetes uncertain’ group may also have diabetes and the remainder may have a pre-diabetic condition, we excluded all from consideration in this study. Our algorithm to differentiate type of diabetes was not useful to exclude participants with type 1 diabetes from the study.

The strength of this study is the large population based sample and the unit record linkage to administrative data collections on claims for occasions of care and events such as hospitalisation. Although a low response rate to invitation to participate was observed, research has demonstrated that for a broad range of risk factors, this study yielded consistent estimates of exposure-outcome relationships as a population survey of the same population that reported varying response rate, sampling frame and mode of questionnaire administration [20]. The main short coming of this study is the absence of the results of diagnostic or other clinical findings to confirm a diagnosis of diabetes among participants. The study relied on responses to a general question ‘*Has a doctor EVER told you that you have diabetes?’* as the primary means of identifying those participants with diabetes. As indicated from additional information available to this study diabetes status could be under-reported through not responding to this question or over-reported in circumstances where diabetes was not longer present for example, gestational diabetes or weight loss. This would not fully explain differences between the different data sets. The questions included in the baseline 45 and Up Study questionnaire did not enable further exploration of diabetes status through seeking additional specific information on diagnosis and care received [21]. Although the administrative data extracts included data collected over a number of years, we have presented a cross-sectional analysis for this study.

Our approach to exploring the reliability of self-report of diabetes was to compare self-report to evidence of a diagnosis of diabetes or diabetes-related testing in the linked data collections. Identification using APDC excluded those patients who were not admitted during data (2000–2009). We found that 80% of participants self-reporting diabetes had a diagnosis of diabetes recorded in APDC; both sensitivity and specificity were high. Using claims for HbA1c testing may over-identify participants with diabetes due to the misuse of this test for screening and other purposes. This was confirmed with lower sensitivity (64%) and Kappa (0.7) which improved to 80.4% and 0.75 when claims for receipt of two HbA1c tests were considered. However, for the diabetes group identified at baseline, our study found that 85% had a record of one or more HbA1c test (73.4% had two or more), confirming the self-report of diabetes in this group. Although it has been suggested that ‘coning’ may lead to an underestimation of diabetes status [7] and this might explain the lower agreement between baseline 45 and Up data and claims for HbA1c testing, we could not explore this in the available data. In regard to our comparison to claims for diabetes related medications recorded in the PBS data, 71% of participants with diabetes had at least one claim for medication in the 12 months prior to recruitment. We observed a high level of agreement (Kappa: 0.8). One reason that only 71% of participants had a prescription for diabetes related medication might be because some medications may cost less than the patient co-payment and thus may not have been recorded in PBBS data during the study period. Some participants are also likely to be controlled by diet alone although we could not demonstrate this in the current data. However, self-reported diabetes medication (Insulin: 10%; OHAs: 58%) was similar to that observed in the PBS data (71%).

Our algorithm identified a small group of participants whose diabetes status was classified as uncertain. These included 136 participants who did not use the tick box but wrote words for conditions such as pre-diabetes or diabetes insipidus and 1,246 participants who did not self-report diabetes but who did report use of oral hypoglycaemic agents. Using additional data that was available through linkage, it is likely that about half of these participants actually have a diagnosis of diabetes that was incorrectly reported at baseline.

The recruitment questionnaire did not seek information on type of diabetes. We attempted to use additional data provided within the questionnaire to identify those participants who had type 1 diabetes. Our algorithm could identify less than half of participants with type 1 diabetes identified in the APDC. As these data do not include all participants in the 45 and Up Study (74% of participants had a hospital admission identified in the 10 year period), it was not possible to use APDC data as an alternate source of diagnosis.

We have identified some differences in reporting of diabetes status between these data collections. Firstly, self-report accurately identified at least 80% of participants in the 45 and Up Study with a diagnosis of diabetes. This is similar to the proportion who self-identified in the AusDiab study where self-report of diabetes was confirmed by biological testing (unpublished data) and suggests that the general question relating to diabetes status was adequate to identify a sub-population of people with diabetes participating in the 45 and Up Study. We are not able to comment on the implications of the findings of the AusDiab study that about half of people with diabetes were not diagnosed at testing. While some under-diagnosis is suggested from the APDC where 16% of participants identified with diabetes on admission did not self-report a diagnosis of diabetes, 21% of participants who self-reported a diagnosis at baseline did not have a diagnostic code for diabetes in their admission data.

Although HbA1c testing is intended for monitoring control of blood sugars among people with diabetes, the low sensitivity and specificity of HbA1c from a single test, that improved when limited to two or more tests, limits the value of this test in identifying a population sample of people with diabetes from the universal health insurance data base. We did not explore the role of other tests, such as diabetes service incentive payments (SIP) for completion of an annual cycle of diabetes care, as these were only claimed for 25% of our diabetic study population [22]. Efforts to create a ‘pseudoSIP’ based on claims for components of the annual cycle of care were not consistent with claims for SIP and were not pursued. These results have implications for national data systems using this means of identification of the incidence and prevalence of diabetes; in particular, the use of the HbA1c test as an indicator of a diagnosis of diabetes is increasingly unsatisfactory due to changes in the eligibility criteria for testing and its more widespread use as a screening test for diabetes. In turn, there are implications for the Australia Medicare Diabetes Practice Incentive Payment which uses the number of HbA1c test claimed to determine the denominator for calculating the proportion of standardised patient equivalents that have completed their annual cycle of care.

Overall the use of survey and administrative data collections pose challenges in identifying community based populations with diabetes; self-report may under- or over-estimate diabetes prevalence; APDC depends on admission and recording of diabetes if not directly related to the admission; and MBS and PBS claims depend on the availability of claims for specific tests.

## Conclusion

This study formed part of preparatory work in a larger research project to identify participants within the 45 and Up Study with a diagnosis of diabetes. A combination of self-reported diabetes augmented with free text data in which a participant reported diabetes as a chronic condition and/or use of insulin among medications used was able to identify participants with diabetes with high sensitivity and specificity compared to available administrative data collections. Using these methods, the diabetes status of a number of participants was found to be uncertain; we have made a decision to exclude these participants from further study. We developed an algorithm to classify participants’ diabetes status into type of diabetes but, due to insufficient information, this was found to be not reliable.

## Competing interests

The authors declare that they have no competing interests.

## Authors’ contributions

EC is the study leader. She conceptualised the study, led the design and implementation of the study, oversaw data analysis and wrote this manuscript. **DT** was employed as the project officer. She participated in the implementation of the study, performed the statistical analysis and helped to draft the manuscript. MH is a senior member of the research group. She provided expert advice on economic aspects of the research. She was a member of the study advisory group and has made a significant contribution to the preparation of this paper. JF is a senior member of the research group. He has provided advice on the development of the research, classification of the study variables, and contributed to the implementation of the study. BJ is a senior member of the research team and has contributed to the development and implementation of the study and the preparation of this manuscript. **LJ** is a senior member of this research group and is a member of the 45 and Up Study Research Team. She has contributed to the development and implementation of the research and contributed to the preparation of this manuscript. MFH is a senior member of the research group. He has contributed extensively to the study design, implementation, and interpretation of the analysis and writing of the manuscript. All authors read and approved the final manuscript.

## Pre-publication history

The pre-publication history for this paper can be accessed here:

http://www.biomedcentral.com/1472-6963/13/481/prepub
